# Transient short-chain fatty acid exposure and the temporal question of epithelial barrier immunity

**DOI:** 10.3389/fimmu.2026.1930311

**Published:** 2026-09-24

**Authors:** Rebecca Lewandowski

**Affiliations:** Department of Molecular and Cellular Biology, University of Arizona, Tucson, AZ, United States

**Keywords:** chromatin, epithelial barrier, epithelial memory, microbiome, mucosal immunity, postbiotics, probiotics, short-chain fatty acids

## Abstract

Studies of microbiome-targeted interventions often use persistence of an administered organism or endpoint metabolite abundance as markers of biological consequence, but host barriers may be altered by microbial chemistry after the initiating exposure has disappeared. Short-chain fatty acids (SCFAs), including acetate, propionate, and butyrate, provide a tractable model for this temporal problem because they connect microbial fermentation to epithelial metabolism, G protein-coupled receptor signaling, tight-junction regulation, immune tone, and chromatin-associated gene regulation. This Perspective argues that SCFA biology should be used to distinguish three outcomes at the host-microbial interface: exposure-dependent modulation, reversible epithelial reprogramming, and durable barrier imprint. Current evidence supports robust epithelial responses to SCFAs and makes persistence biologically plausible through chromatin-associated regulation and epithelial immune memory, but it does not yet establish whether brief adult SCFA exposure can alter later barrier behavior after withdrawal. Clarifying this conversion step will provide a common temporal framework for dietary, prebiotic, probiotic, postbiotic, and microbiota-transfer studies by distinguishing intervention delivery and engraftment from epithelial persistence.

## Introduction

The most unresolved consequences of a microbiome-targeted intervention may begin after its administered organism, substrate, or metabolite is gone. If an introduced microbe fails to engraft and the metabolite pulse it produced fades, the host barrier is often presumed to have returned to its prior state. That presumption is incomplete because it equates loss of microbial residence with loss of host consequence. A transient organism may still produce metabolites, alter local chemistry, interact with resident microbial networks, or change epithelial exposure before it is cleared.

Probiotic engraftment is an ecological outcome shaped by resident community structure, niche availability, substrate access, host physiology, and mucosal chemistry. Empiric probiotic administration produces person-specific mucosal colonization patterns, and colonization resistance can limit engraftment in humans (1). Engraftment may extend or renew metabolite exposure when relevant dietary substrates are available (1–3), but it does not alone establish metabolite production, epithelial exposure, or persistence of the host response.

The central question is therefore not only whether an introduced organism persists, but whether a transient microbial metabolite can generate epithelial behavior that outlasts the exposure that produced it. This is an immunological question because epithelial barriers are not passive surfaces. Intestinal epithelial cells participate in antigen presentation and respond to immune-derived cytokines that regulate epithelial renewal and differentiation. For example, subsets of intestinal stem cells express major histocompatibility complex class II machinery and interact with CD4 T cells, linking epithelial maintenance to mucosal immune responses (4). A transient metabolite that changes epithelial barrier behavior may therefore alter how mucosal tissue interprets later microbial, inflammatory, or dietary encounters, even when the original organism is no longer detectable.

Short-chain fatty acids (SCFAs) are the most useful model for this question. Acetate, propionate, and butyrate are not the only microbial products capable of shaping epithelial or immune biology. Indoles, bile acid derivatives, polyamines, purines, phenolic metabolites, peptidoglycan fragments, lipopolysaccharide, microbial vesicle cargo, and oxygen-linked metabolic shifts also participate in host-microbial communication (5, 6). SCFAs are especially tractable because they are abundant, inducible, experimentally manipulable, and mechanistically connected to epithelial energy metabolism, G protein-coupled receptor signaling, histone deacetylase activity, tight-junction regulation, immune tone, and chromatin-associated gene regulation (2, 3, 7). Relevant receptors include free fatty acid receptor 2 (FFAR2/GPR43) and free fatty acid receptor 3 (FFAR3/GPR41), which respond to SCFAs, and hydroxycarboxylic acid receptor 2 (HCAR2/GPR109A), which responds to butyrate (8, 9).

This Perspective uses SCFAs to separate three outcomes that are often collapsed in studies of microbiome-targeted interventions. The first is exposure-dependent modulation, in which the epithelial effect depends on continued metabolite exposure. The second is reversible epithelial reprogramming, in which epithelial state changes after exposure but returns to baseline without altering later challenge responses. The third is durable barrier imprint, in which prior exposure changes how the epithelium responds after withdrawal, especially after inflammatory, microbial, or injury-associated challenge. This distinction makes the temporal claim testable. It separates the question of whether the barrier responded from the stronger question of whether the barrier was changed.

## SCFAs as ecological products and epithelial inputs

SCFAs are both ecological products of microbial fermentation and direct inputs into epithelial physiology. Their abundance reflects the interaction of dietary substrate, resident fermentative capacity, cross-feeding, transit time, antibiotic exposure, and intestinal region (2, 3, 10). Repeated dietary inputs may therefore renew metabolite exposure, although the resulting concentration, timing, and epithelial compartment remain conditional (2, 3). The same molecules can be absorbed, metabolized, and sensed by epithelial and immune cells, making an SCFA an ecological readout, metabolic substrate, and immunologic modifier depending on where and when it is encountered (3, 7).

This dual identity also creates a persistent interpretive challenge. Detection in stool or lumen establishes metabolite presence, but does not by itself establish its source, production rate, or the epithelial concentration, timing, spatial distribution, duration, or cellular compartment of exposure. Luminal detection is not equivalent to exposure at the mucus-associated surface, crypt-adjacent region, regenerative niche, or immune-epithelial interface. Epithelial consequence therefore cannot be inferred from metabolite abundance alone. For a transient metabolite to matter beyond the exposure interval, the field must show not only that epithelial cells encountered the molecule, but that the encounter changed epithelial behavior during or after the signal passed.

The evidence for epithelial response is substantial, even if the evidence for post-exposure persistence remains less developed. Across several models, SCFAs affect multiple layers of barrier biology. First, SCFAs can alter the physical architecture of the epithelial barrier. Butyrate enhances barrier function in Caco-2 monolayers by promoting tight-junction assembly through AMP-activated protein kinase-associated signaling (11). Related work shows that butyrate can increase expression of claudin-1, a tight-junction protein that supports barrier sealing, and that loss of claudin-1 reduces the protective effect in that model (12). Butyrate can also repress claudin-2, a pore-forming tight-junction protein associated with increased permeability, through an IL-10 receptor alpha-dependent mechanism (13). These studies show that butyrate can influence not only whether the epithelial barrier is tighter or leakier, but also the junctional architecture through which that change occurs.

Second, SCFA effects are metabolic as well as structural. Butyrate contributes to colonocyte energy metabolism and can regulate autophagy in the mammalian colon (14). Microbiota-derived SCFAs also support epithelial hypoxia-inducible factor signaling and barrier function, linking microbial fermentation to epithelial oxygen biology and tissue protection (15). Epithelial barrier function therefore emerges from coordinated energy use, oxygen handling, junctional organization, repair programs, and immune-facing communication rather than from tight-junction structure alone.

Third, epithelial responses differ by metabolite identity and concentration. In human induced pluripotent stem cell-derived intestinal epithelial layers, 24-hour exposure to acetate, propionate, or butyrate produces distinct whole-genome expression profiles, with butyrate producing the strongest transcriptional response, propionate producing an intermediate response, and acetate producing a weaker response in a concentration-dependent manner (16). This moves the field away from a generic SCFA effect and toward a more precise question of which metabolite reaches which epithelial compartment, at what dose, for how long, and in what host state.

## The chromatin turn

A major recent shift is that SCFAs are no longer understood only as microbial fermentation products, epithelial metabolic substrates, or short-term modifiers of barrier physiology. They are now implicated in chromatin-associated regulation, which changes the kind of question the field can ask. A metabolite that affects tight-junction function may explain an acute barrier response, whereas one linked to chromatin state raises the possibility that microbial chemistry influences future epithelial gene regulation.

Recent *in vivo* work provides the first layer of this argument. Histone butyrylation and propionylation are detectable in the mouse intestinal epithelium, are shaped by the microbiota, and are associated with epithelial gene-expression programs (17). Tributyrin, a triglyceride form of butyrate used experimentally to increase butyrate availability, further links this chemistry to epithelial regulation. In mouse intestinal epithelium, tributyrin altered gene-expression programs, and those changes correlated with histone butyrylation at active gene-regulatory elements (17). Together, these findings connect microbial metabolite availability to epithelial chromatin regulation in intact tissue, rather than only in simplified exposure systems.

A second layer makes the mechanism more specific. Propionate and butyrate have been identified as contributors to short-chain acyl-lysine histone marks, including propionylation and butyrylation at H3K18 and H4K12 (18). These marks have been mapped in normal cells, colorectal cancer cells, and mouse intestines, where they are associated with chromatin accessibility and genes involved in growth, differentiation, and ion transport (18). Butyrate and propionate can also inhibit histone deacetylase activity, as demonstrated in experimental assays (19). The histone acylation findings therefore broaden the mechanisms connecting SCFAs to chromatin regulation (17, 18), without establishing persistence after withdrawal.

These studies do not prove that brief SCFA exposure creates durable epithelial memory, and that limitation is central to their interpretation. The current chromatin evidence establishes that SCFAs can intersect with epithelial regulatory systems during exposure, sustained substrate availability, or endpoint measurement. It does not establish whether those chromatin-associated changes persist after the metabolite source is removed, or how long any persisting changes remain detectable. If SCFA-linked chromatin changes remain dependent on ongoing exposure, they support acute or reversible epithelial modulation. If some changes persist after the metabolite pulse has ended and alter later epithelial challenge responses, they would support a stronger model in which transient microbial chemistry changes future barrier behavior. The current chromatin literature therefore makes durable imprint mechanistically plausible but not yet demonstrated.

## Response is not the same as future behavior

The chromatin evidence brings SCFAs closer to epithelial state regulation, but it does not resolve the temporal problem. A chromatin-associated change measured during exposure, after supplementation, or at a single endpoint may indicate that epithelial cells entered a different regulatory state. It does not show whether that state persisted, resolved, or changed a later response after the metabolite source was removed. **Figure 1** summarizes exposure-dependent modulation, reversible epithelial reprogramming, and durable barrier imprint according to the timing of withdrawal and subsequent assessment.

**Figure 1:**
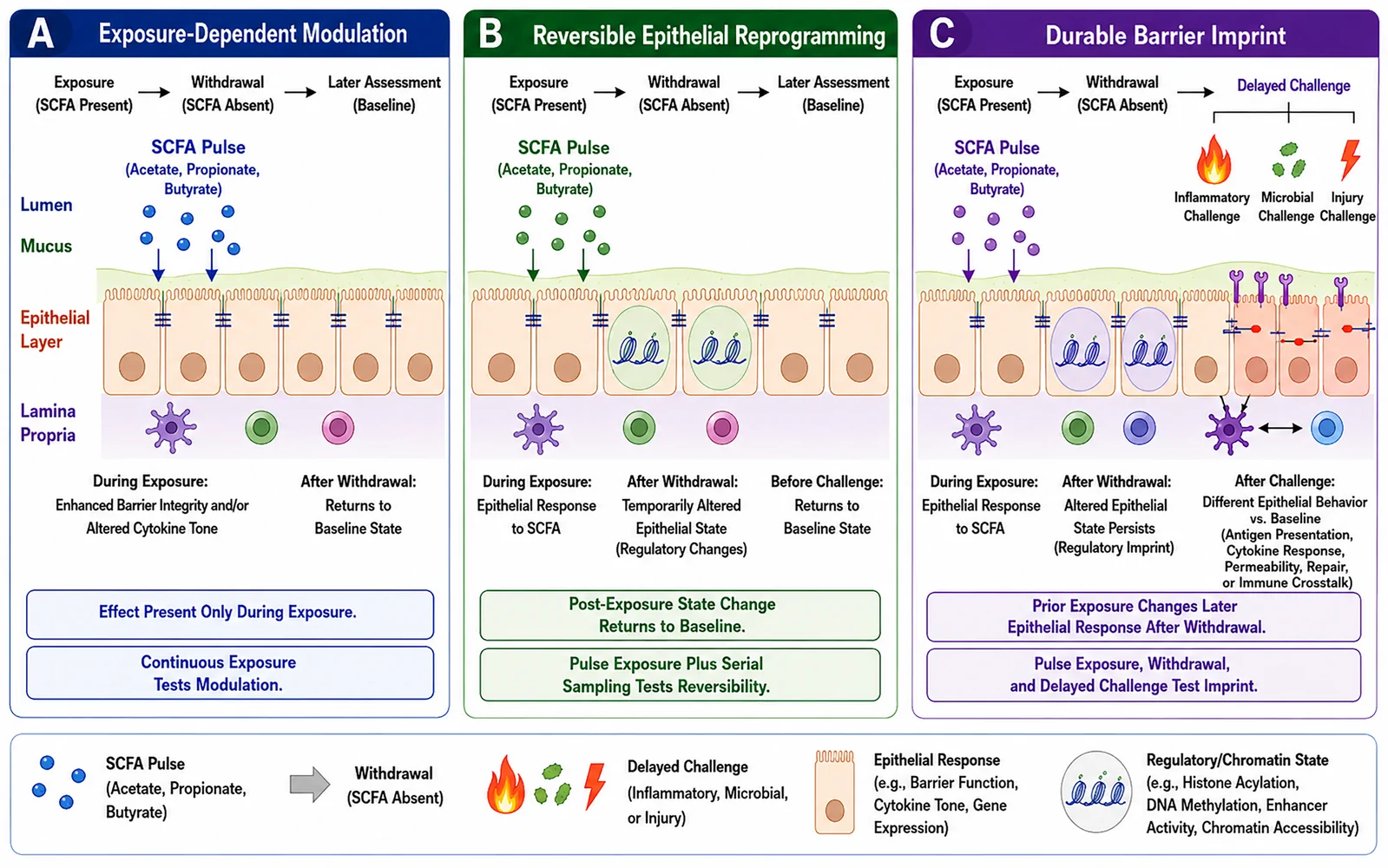
Temporal outcomes after transient SCFA exposure. Transient exposure to short-chain fatty acids (SCFAs) can support at least three distinct biological claims. In exposure-dependent modulation, the epithelial effect depends on continued metabolite exposure and returns to baseline after that exposure ends. In reversible epithelial reprogramming, epithelial state changes after exposure but returns to baseline without altering later challenge responses. In durable barrier imprint, prior exposure changes epithelial behavior after withdrawal, especially after inflammatory, microbial, or injury-associated challenge. Separating the exposure phase from the withdrawal and delayed test phases is necessary to distinguish epithelial response from epithelial memory. Three-panel schematic showing temporal outcomes after transient short-chain fatty acid exposure. **(A)** shows exposure-dependent modulation, with an epithelial effect present during SCFA exposure and return to baseline after withdrawal. **(B)** shows reversible epithelial reprogramming, with a transient post-exposure epithelial state change that returns to baseline before later assessment. **(C)** shows durable barrier imprint, with prior SCFA exposure followed by withdrawal and delayed inflammatory, microbial, or injury challenge, resulting in altered epithelial behavior compared with baseline.

The stronger claim requires a different experimental logic. A barrier improvement during butyrate exposure does not establish that a later inflammatory insult will be handled differently after butyrate is removed. A 24-hour transcriptional response does not establish a persistent epithelial operating state. A histone acylation mark associated with gene regulation does not establish that the mark persists after withdrawal or that it changes later barrier function. In each case, the missing evidence is whether the response altered what the epithelium does next.

Epithelial renewal makes persistence a compartment-specific question. A claim of cell-intrinsic memory across renewal requires evidence that an altered regulatory state is retained within a self-renewing epithelial lineage. Persistent chromatin accessibility after inflammation has been demonstrated in mouse skin epithelial stem cells (20), while early-life intestinal microbial signaling provides an example involving stem-cell DNA methylation and later epithelial immune function (21). These findings motivate tests of chromatin accessibility, enhancer regulation, and DNA methylation as candidate mechanisms; they do not establish that brief adult SCFA exposure engages them. Persistence maintained by immune or stromal feedback should be distinguished from epithelial cell-intrinsic memory, and repeated SCFA exposure from persistence after withdrawal.

Recent work on early-life microbial signaling illustrates what persistence can look like while also defining the boundary of inference. In mice, microbial cues around the weaning transition shape DNA methylation and transcription in intestinal stem cells through an interferon gamma pathway, producing an epithelial major histocompatibility complex class II state that persists into adulthood (21). That state is immunologically meaningful because it changes how the barrier participates in antigen presentation, immune surveillance, and communication with CD4 T cells. Blocking interferon gamma during early life disrupted this microbiome-mediated effect, whereas blockade in adulthood did not reproduce the same result (21). This is not evidence that brief SCFA exposure in an adult epithelium can independently produce the same kind of imprint. It shows that epithelial memory is possible when a signal reaches a receptive compartment and is coupled to regulatory mechanisms capable of surviving epithelial renewal.

The test of epithelial memory is therefore not whether SCFAs changed the epithelium during exposure, but whether prior exposure changes the epithelial response to a later challenge after withdrawal.

## The conversion problem

For SCFAs, the conversion problem is what determines whether a metabolite pulse remains exposure-dependent modulation, becomes reversible epithelial reprogramming, or produces durable barrier imprint. Conversion is likely governed by dose, compartment, timing, and epithelial state. **Table 1** summarizes the experimental evidence needed to distinguish SCFA detection, epithelial exposure, exposure-dependent modulation, reversible epithelial reprogramming, durable barrier imprint, and stem or progenitor-associated imprint.

**Table 1:** Evidence needed to distinguish SCFA response from epithelial memory.

Claim	Required design	Minimum readout	What the claim cannot infer
*SCFA Detection*	Stool, luminal, tissue, or metabolomic measurement	Acetate, propionate, butyrate, or related metabolite concentration	Epithelial exposure, spatial distribution, or functional consequence
*Epithelial Exposure*	Compartment-resolved sampling or validated epithelial exposure model	Concentration and timing at mucus-associated, crypt-adjacent, surface, or immune-epithelial interface	Persistence after withdrawal
*Exposure-Dependent Modulation*	Continuous or acute SCFA exposure with epithelial readout during exposure	Barrier function, tight-junction expression, transcription, metabolism, cytokine response, or chromatin-associated mark	Future epithelial behavior after exposure ends
*Reversible Epithelial Reprogramming*	Pulse exposure, withdrawal, and serial post-exposure sampling	Temporary epithelial state change after exposure with return to baseline	Altered response to later inflammatory, microbial, or injury challenge
*Durable Barrier Imprint*	Pulse exposure, verified withdrawal, serial follow-up, and delayed challenge, with previously exposed and unexposed groups assessed with and without challenge	Altered post-challenge barrier function after the initiating exposure has ended, with residual exposure and unresolved injury assessed	Whether the imprint is beneficial, harmful, compartment-specific, or reversible
*Stem or Progenitor-Associated Imprint*	Pulse exposure, verified withdrawal, and lineage-resolved analysis across documented epithelial renewal	Retained regulatory state and altered function in lineage descendants, with proliferation, viability, and regenerative capacity measured	Beneficial adaptation, absence of clonal selection, or transferability across developmental, homeostatic, and inflammatory settings

SCFA, short-chain fatty acid. The categories shown are not mutually exclusive biological states; they represent progressively stronger evidentiary claims about the temporal relationship between metabolite exposure and later epithelial behavior. These evidentiary categories apply whether SCFA exposure arises from diet, prebiotics, probiotics, postbiotics, or microbiota transfer. Across-renewal persistence does not alone establish cell-intrinsic inheritance; sustained niche signals and selection of pre-existing cell states must also be considered.

SCFA effects depend on the compartment exposed. It has been shown that differentiated colonocytes consume butyrate and help protect crypt stem/progenitor cells; direct exposure inhibited proliferation through a mechanism involving histone deacetylase inhibition and Foxo3, and impaired repair in injury settings (22). Physiological luminal concentrations therefore cannot be assumed to represent physiological stem-cell exposure. Any proposed stem-cell imprint would require testing whether a compartment-specific exposure alters later epithelial behavior and whether that persistence is accompanied by impaired proliferation or regeneration. Indirect signaling through immune or niche cells is another candidate route, given evidence that immune-derived cytokines regulate intestinal stem-cell behavior (4), but its mediation of SCFA-induced memory remains unproven.

Context dependence also makes bulk SCFA measurement an insufficient proxy for epithelial reprogramming. The relevant exposure is spatially specific. An SCFA detected in stool, luminal contents, or whole-tissue extracts does not establish what concentration reached the mucus-associated surface, crypt-adjacent region, regenerative niche, or immune-epithelial interface. Bulk measurements collapse concentration, timing, intestinal region, microbial source, diffusion distance, uptake, and epithelial compartment into a single endpoint. Without spatial resolution, it is difficult to determine whether SCFA exposure reached a compartment capable of converting transient signal into later epithelial behavior.

## What durable barrier imprint would mean

Exposure-dependent modulation may require repeated dosing or sustained substrate availability. A response that persists after withdrawal raises different questions about its duration, cellular location, mechanism, and effect on later challenge.

This distinction also changes how benefit and risk should be interpreted. A reversible increase in barrier integrity during inflammation has different implications than a durable change in epithelial antigen presentation, cytokine responsiveness, permeability, or regenerative behavior. A response confined to differentiated epithelial cells has different implications than one that reaches stem or progenitor compartments capable of carrying regulatory information through epithelial renewal. A transient metabolite exposure in an adult steady-state barrier is not equivalent to exposure during development, injury, immune compromise, or active inflammatory disease.

Gut epithelial disease models support this risk-benefit logic. In Crohn’s disease, a sustained inflammatory secretory progenitor state has been identified in the intestinal epithelium, with accessible chromatin in disease-associated stem cells and persistent inflammatory gene expression in patient-derived colonoids (23). This is not evidence that SCFAs create the same state. It shows why durable epithelial states change the meaning of epithelial response. A lasting epithelial program may influence repair, inflammatory responsiveness, disease progression, or therapeutic vulnerability, depending on which compartment carries the altered state and what signals amplify it later.

For this reason, durability is not automatically desirable. A durable epithelial effect could support resilience, but it could also alter immune surveillance, tolerance, repair, or inflammatory thresholds in ways that depend on host context. The central issue is therefore not simply whether SCFA-linked changes endure after exposure. It is what kind of epithelial future they create, in which compartment, under what conditions, and with what trade-offs.

## Discussion and future directions

Whether brief adult SCFA exposure alters later barrier behavior remains unresolved. The next priority is to distinguish residual exposure, transient recovery, and persistent changes in challenge responsiveness.

Adult intestinal organoids and colonoids should undergo defined SCFA pulses followed by verified washout across documented cell divisions and serial passages, with unexposed and continuously exposed controls. Patient-derived colonoids already provide a model for persistent epithelial states (23), while primary intestine-on-chip systems offer controlled perfusion and compartmental access (24). For SCFA experiments, regional identity and exposure polarity should match the biological question, and washout should be measured rather than inferred from medium replacement. Delayed inflammatory or microbial challenge should test permeability, cytokine responses, and repair alongside viability and proliferation.

Durability should be reported over a defined observation interval rather than treated as permanence. Several post-withdrawal challenge times would distinguish a slowly resolving response from an imprint that remains detectable across epithelial renewal. At each interval, previously exposed and unexposed cultures should be assessed both with and without challenge, allowing persistent baseline differences to be separated from altered challenge responsiveness. A return to baseline before challenge would not exclude a latent imprint, whereas an altered post-challenge endpoint alone could reflect a pre-existing difference. These comparisons would establish whether prior exposure changes the response to challenge beyond its effect on the pre-challenge epithelial state.

Mechanistic studies should combine lineage tracking with serial chromatin-accessibility profiling, histone-mark mapping, and DNA-methylation analysis. Cleavage under targets and tagmentation (CUT&Tag) offers a low-input approach to chromatin profiling (25); assays of SCFA-associated acylation require validated mark-specific reagents. Measurements before exposure, after washout, and in lineage descendants should be paired with functional testing. Serial detection alone cannot distinguish inherited regulation from clonal selection or continued niche signaling. Perturbing candidate regulators after withdrawal would test whether they maintain the altered response. Epithelial-only cultures and matched immune or stromal cocultures could distinguish cell-intrinsic persistence from feedback-dependent effects.

Diet, probiotic, and postbiotic trials should prespecify post-intervention follow-up and distinguish cessation of administration from cessation of the resulting exposure. Dietary substrate and microbial production can sustain SCFA availability (2, 3, 10, 26). Because endogenous production continues, human studies should assess whether intervention-associated exposure has returned toward baseline rather than require complete SCFA absence. Clinical outcomes and barrier-function measures should be assessed alongside exposure, with biopsy-derived epithelial assays where feasible. Persistent benefit alone would not establish epithelial memory. The framework’s translational value is to determine whether continued dosing is needed, how long an effect lasts, and whether persistence preserves repair or sustains an adverse epithelial state.

## Data Availability

The original contributions presented in the study are included in the article/supplementary material. Further inquiries can be directed to the corresponding author.
